# Impact of diabetes mellitus on drug-resistant tuberculosis across resistance categories: a systematic review and meta-analysis

**DOI:** 10.7189/jogh.16.04170

**Published:** 2026-05-12

**Authors:** Boya Gou, Jin Wang, Han Liu, Shuihua Lu, Xiaomin Wang

**Affiliations:** 1 National Clinical Research Centre for Infectious Diseases, Shenzhen Third People's Hospital, Shenzhen, China; 2 Department of Internal Medicine, Shenzhen University Health Science Centre, Shenzhen University, Shenzhen, China

## Abstract

**Background:**

Tuberculosis (TB) and diabetes mellitus (DM) are major global health challenges that frequently coexist. DM has been associated with adverse TB outcomes, but its relationship with specific categories of drug-resistant TB has not been comprehensively synthesised. The association between DM and different drug-resistance categories in patients with TB was quantified, with a primary focus on resistance to first-line anti-TB drugs.

**Methods:**

A systematic review and meta-analysis of observational studies was conducted, drawing on evidence identified through seven international and Chinese databases, in accordance with the PRISMA (Preferred Reporting Items for Systematic Reviews and Meta-Analyses) guidelines. Study quality was assessed using the Newcastle-Ottawa Scale and the Agency for Healthcare Research and Quality checklist. Random-effects models were used to pool odds ratios (ORs) and 95% confidence intervals (CIs).

**Results:**

Forty-five studies involving 51 982 participants were included. Compared with patients without DM, those with DM had higher odds of isoniazid-resistant TB (OR = 1.30; 95% CI = 1.14–1.48), rifampicin-resistant TB (OR = 1.37; 95% CI = 1.17–1.59), and multidrug-resistant TB (OR = 1.45; 95% CI = 1.12–1.88). Associations for polydrug-resistant TB and extensively drug-resistant TB were imprecise and were based on relatively few studies. Subgroup analyses suggested possible geographic variation, although the evidence base was heavily concentrated in East Asia. Exploratory analyses of studies reporting glycaemic data suggested that poorer glycaemic control may be associated with higher odds of drug resistance, but definitions and thresholds were inconsistent across studies.

**Conclusions:**

DM was associated with higher odds of isoniazid-resistant TB, rifampicin-resistant TB, and multidrug-resistant TB. These findings should be interpreted as associations rather than evidence of causality, but they support closer integration of TB and DM assessment in clinical care and public health practice.

Diabetes mellitus (DM) and tuberculosis (TB) remain two major global public health challenges with substantial and increasingly overlapping burdens [1,2]. A bidirectional relationship between the two conditions has been widely recognised. Compared with people without DM, those with DM have an approximately two- to 3-fold higher risk of developing active TB [3,4]. Several biological mechanisms may underlie this association. Hyperglycaemia can impair both innate and adaptive immune responses by reducing macrophage and neutrophil phagocytic function, limiting chemotaxis, and weakening T-cell activation, thereby creating a permissive environment for the persistence of *Mycobacterium tuberculosis* [5]. In the opposite direction, TB disease and anti-TB treatment may disrupt glucose metabolism, worsen glycaemic control, and complicate diabetes management [6]. In addition, chronic metabolic derangements associated with DM may alter the pharmacokinetics of key anti-TB drugs, particularly rifampicin, and may adversely affect treatment response [7,8].

According to the Global Tuberculosis Report 2025, an estimated 10.7 million people developed TB and about 1.23 million died from the disease worldwide in 2024 [1]. The latest International Diabetes Federation estimates indicate that 589 million adults aged 20–79 years were living with DM in 2024, and this number is projected to rise to 853 million by 2050 [2]. Most people living with DM reside in low- and middle-income countries, where high TB endemicity often coexists with constrained access to diabetes prevention, diagnosis, and long-term care [2,3]. This convergence of epidemics creates major challenges for clinical management and public health control, particularly in settings with fragile laboratory capacity and limited resources for integrated care [1,2].

The growing burden of drug-resistant TB adds another layer of complexity. Several systematic reviews and meta-analyses have suggested that DM is associated with higher odds of multidrug-resistant TB (MDR-TB), defined as resistance to at least isoniazid and rifampicin [9-12]. However, the available evidence remains heterogeneous across study designs, populations, and health-system contexts. Such heterogeneity may reflect differences in case mix, implementation of directly observed treatment programmes, access to drug susceptibility testing, prior treatment history, and underlying host or pathogen characteristics [13,14]. Mechanistic and pharmacokinetic evidence also supports the plausibility of this association. Hyperglycaemia-related immune dysfunction may favour the emergence or persistence of resistant strains, while reduced drug exposure in people with DM may further compromise microbiological response during treatment [5,7,8,13]. Isoniazid monoresistance is also clinically important because it may represent an early step in the pathway towards more advanced resistance phenotypes [15]. Furthermore, poorer glycaemic control, commonly assessed using glycated haemoglobin, has been associated with less favourable TB outcomes and may also be related to higher odds of drug resistance [6,16].

Despite these findings, most existing evidence syntheses have focused predominantly on MDR-TB, with much less attention given to other resistance categories, including isoniazid resistance, rifampicin resistance, polydrug-resistant TB, and extensively drug-resistant TB [9-12]. In addition, the potential modifying effects of glycaemic status and previous TB treatment have not been consistently examined across different populations. These gaps limit a more comprehensive understanding of how DM may be associated with the full spectrum of TB drug resistance and constrain the development of integrated, evidence-based strategies for screening, risk stratification, and clinical management. To address these gaps, we conducted a systematic review and meta-analysis.

The objectives of this study were:

(a) to quantify the prevalence of drug-resistance patterns across various resistance categories among patients with TB with and without DM

(b) to determine the odds of drug resistance associated with TB-DM comorbidity

(c) to explore whether glycaemic control and previous TB treatment modified the association between DM and drug resistance.

By addressing these gaps, this study aimed to provide evidence to inform more targeted screening strategies, support risk stratification, and strengthen integrated approaches to the management of patients affected by both TB and DM.

## METHODS

### Study protocol and registration

This systematic review and meta-analysis were conducted in accordance with the Preferred Reporting Items for Systematic Reviews and Meta-Analyses (PRISMA) 2020 statement [17]. The study protocol was prospectively registered in the International Prospective Register of Systematic Reviews (PROSPERO; registration number CRD420251089146).

### Search strategy and information sources

The full search strategies for each database are provided in Supplementary Material 1 in the **Online Supplementary Document**. The search covered publications from 1 January 1998 to 1 July 2025. The search strategy employed a combination of Medical Subject Headings terms. Additionally, we used keywords combined with ‘AND’ and ‘OR’ Boolean operators to ensure sufficient and comprehensive results. Key search terms included ‘diabetes mellitus’, ‘tuberculosis’ OR ‘TB’ OR ‘pulmonary tuberculosis’, and ‘drug resistance, microbial’ OR ‘drug-resistant’ OR ‘drug resistance, bacterial’. 

### Eligibility criteria and study selection

The inclusion of observational studies was undertaken, with the inclusion of cohort, case-control, and cross-sectional studies, that enrolled participants with microbiologically or clinically confirmed TB and reported at least one drug-resistance outcome relevant to this review. Eligible outcomes included resistance to specific anti-TB drugs and recognised resistance categories, such as isoniazid-resistant TB, rifampicin-resistant TB, multidrug-resistant TB, polydrug-resistant TB, and extensively drug-resistant TB.

For comparative association analyses, studies were required to provide sufficient data to compare participants with and without DM, either through directly reported effect estimates or extractable data for constructing 2 × 2 tables. Studies reporting data for only one glycaemic or DM stratum were retained only when they contributed relevant prevalence data to descriptive or exploratory syntheses. We excluded reviews, editorials, letters, case reports, animal or laboratory studies, and conference abstracts or proceedings that did not provide sufficient methodological detail or extractable outcome data.

All retrieved records were imported into EndNote, version X21 (Clarivate, Philadelphia, PA, USA), and duplicates were removed. Two reviewers (BYG and JW) independently screened titles and abstracts and subsequently assessed full-text articles for eligibility. Disagreements were resolved through discussion and, when necessary, consultation with a third reviewer (HL).

### Data extraction and quality assessment

Two reviewers (BYG and JW) independently extracted data using a piloted standardised form. Extracted variables included first author, publication year, country or region, study design, diagnostic criteria for TB and DM, total sample size and group distribution, previous TB treatment history, drug susceptibility testing methods, and the frequency of drug resistance by type. Inter-reviewer agreement for data extraction was high (kappa >0.85). Any discrepancies were resolved through discussion and adjudication by a third reviewer (HL).

Methodological quality was assessed using the Newcastle-Ottawa Scale (NOS) for cohort and case-control studies and the Agency for Healthcare Research and Quality (AHRQ) checklist for cross-sectional studies [18,19]. For NOS, scores of 7–9 were considered high quality, 5–6 moderate quality, and less than 5 low quality. For AHRQ, scores of 8–11 were considered high quality, 4–7 moderate quality, and 0–3 low quality. All eligible studies were included in the primary analyses, and prespecified sensitivity analyses excluding low-quality studies were conducted to assess the robustness of the pooled estimates.

### Outcome definitions

Outcomes were defined according to the World Health Organization (WHO) guidelines for drug-resistant TB. The following resistance categories were considered:

1. Isoniazid-resistant TB (HR-TB): resistance to isoniazid, with or without resistance to other first-line drugs, excluding rifampicin resistance

2. Rifampicin-resistant TB (RR-TB): resistance to rifampicin, with or without resistance to other drugs

3. MDR-TB: resistance to at least both isoniazid and rifampicin

4. Polydrug-resistant TB (PDR-TB): resistance to more than one first-line anti-TB drug, other than both isoniazid and rifampicin.

5. Extensively drug-resistant TB (XDR-TB): In this review, XDR-TB was defined according to the classification used in each primary study. Most included studies applied the pre-2021 WHO definition (MDR-TB with additional resistance to any fluoroquinolone and at least one second-line injectable agent). We note that the 2021 WHO updated definition reclassifies XDR-TB as MDR/RR-TB with additional resistance to any fluoroquinolone and at least one Group A agent (bedaquiline or linezolid). As the majority of included studies predate this reclassification, the older definition was retained for consistency.

### Statistical analysis

All statistical analyses were performed using Stata, version 18.0 (StataCorp LLC, College Station, TX, USA). Pooled odds ratios (ORs) and 95% confidence intervals (CIs) were calculated using random-effects models based on the DerSimonian-Laird method. Statistical heterogeneity was assessed using Cochran’s Q test and the I-squared (*I*^2^) statistic. For the primary outcomes, 95% prediction intervals were additionally calculated to illustrate the range of effects that might be expected in future comparable studies [20,21].

Publication bias and small-study effects were assessed for meta-analyses with a sufficient number of studies by visual inspection of funnel plots and by Egger’s regression test and Begg’s rank correlation test, with *P* < 0.10 considered indicative of potential asymmetry [22,23]. Because these approaches may perform poorly in the presence of substantial heterogeneity, we also constructed Doi plots and calculated the Luis Furuya-Kanamori index as supplementary assessments [24]. When asymmetry was suspected, the trim-and-fill method was used to explore its potential influence on pooled estimates [25]. For outcomes with relatively low heterogeneity (*I*^2^≤50%), fixed-effect estimates were examined alongside random-effects estimates for comparison.

Prespecified sensitivity analyses included leave-one-out analyses and exclusion of low-quality studies. For outcomes showing substantial heterogeneity, we additionally applied the Hartung-Knapp-Sidik-Jonkman method as a sensitivity analysis to provide a more conservative estimate of uncertainty [26]. Meta-regression analyses were undertaken to explore potential sources of between-study heterogeneity when a sufficient number of studies and adequately reported covariates were available.

Prespecified subgroup analyses were conducted according to study design (cross-sectional *vs*. longitudinal), resistance history (primary *vs*. secondary *vs*. any), geographic region (East Asia, Latin America, USA/Europe, and other regions), DM type (type 2 DM *vs*. unspecified DM), DM ascertainment method (biochemical criteria *vs*. clinical records or self-report), drug susceptibility testing methodology (phenotypic *vs*. molecular), and study period (pre-2010, 2010–2015, 2016–2020, and post-2020).

## RESULTS

### Study screening and characteristics

**Figure 1:**
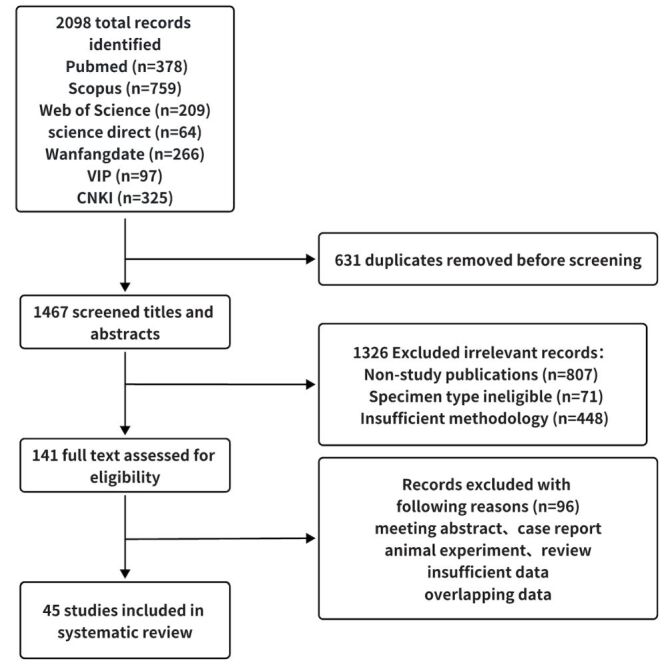
PRISMA 2020 flow diagram of study selection. PRISMA – Preferred Reporting Items for Systematic Reviews and Meta-Analyses.

A total of 2098 records were identified through searches of PubMed (n = 378), Scopus (n = 759), Web of Science (n = 209), ScienceDirect (n = 64), Wanfang Data (n = 266), VIP (Chinese Science and Technology Periodical Database) (n = 97), and CNKI (China National Knowledge Infrastructure) (n = 325). After removing 631 duplicates, 1467 unique records remained for title and abstract screening. Of these, 1326 records were excluded as irrelevant, including non-study publications (n = 807), studies with ineligible specimen types (n = 71), and those with insufficient methodological information (n = 448). Consequently, 141 articles were retrieved for full-text assessment. After detailed evaluation, 96 full texts were excluded for reasons such as conference abstracts, case reports, animal experiments, narrative reviews, insufficient data, or overlapping data sets. Finally, 45 studies met the eligibility criteria and were included in the systematic review. The detailed selection process is illustrated in the PRISMA flow diagram (**Figure 1**).

### Descriptive summary of included studies

Of the 45 included studies, the total sample size comprised 51 982 participants, with individual study sample sizes ranging from 50–21 414 [27–71]. The studies were conducted in various geographic regions: China – including mainland China, Taiwan, and Hong Kong (n = 34), Mexico (n = 3), Georgia (n = 2), and Indonesia, Peru, Ethiopia, the USA, USA/Mexico, and Georgia/Mexico (n = 1 each). The included studies comprised cohort (n = 15), case-control (n = 14), and cross-sectional (n = 16) designs. The majority of studies (n = 19) enrolled patients with type 2 diabetes, whereas the remaining studies either did not specify diabetes type or included mixed diabetes populations. Drug susceptibility testing was performed using phenotypic methods (*e.g*. the proportion method and the Mycobacterial Growth Indicator Tube) in 40 studies and molecular methods (*e.g*. Xpert MTB/RIF (Mycobacterium tuberculosis/rifampicin) and whole-genome sequencing) in five studies. The mean age of participants ranged from 43–69 years across studies. The detailed characteristics of each study are presented in **Table 1**.

**Table 1:** Characteristics of studies included in the meta-analysis

Author, year	Study design	Place		Age	DM type	DR-TB type*	TB patients			NOS/AHRQ†	DM ascertainment‡	DST method§
							**Total**	**DM**	**Not DM**			
Lingdi Lu 1999 [27]	case-control	China	50–60		DM	secondary	61	61	NA	4/9	Biochemical	Phenotypic
Mona Bashar 2001 [28]	case-control	USA	NA		DM	any	155	50	105	9/9	Biochemical	Phenotypic
Bachti Alisjahbana 2007 [29]	cohort	Indonesia	45		T2DM	any	634	94	540	8/9	Biochemical	Phenotypic
SP Fisher-Hoch 2008 [30]	cohort	USA, Mexico	NA		T2DM	any	1442	401	1041	8/9	Clinical	Phenotypic
Chunxiao Zhang 2008 [31]	case-control	China	43–76		T2DM	any	128	56	72	6/9	Biochemical	Phenotypic
Xuguang Shao 2010 [32]	cohort	China	NA		DM	any	961	210	751	4/9	Biochemical	Phenotypic
Jenn-Tyang Chang 2011 [33]	cohort	China (Taiwan)	56.6 ± 12.7		T2DM	any	630	189	441	7/9	Biochemical	Phenotypic
Huiping Duan 2012 [34]	cross-sectional	China	47.5		DM	any	568	54	514	5/11	Biochemical	Phenotypic
AH Hsu 2013 [35]	cross-sectional	China (Taiwan)	NA		DM	any	1008	245	763	8/11	Biochemical	Phenotypic
MJ Magee 2013 [36]	cohort	Peru	NA		DM	any	1671	186	1485	8/9	Biochemical	Phenotypic
Yu Xiong 2013 [37]	cross-sectional	China	61–88		DM	primary	182	88	94	5/11	Biochemical	Phenotypic
Fengling Mi 2014 [38]	cross-sectional	China	NA		T2DM	any	621	187	434	7/11	Biochemical	Phenotypic
Matthew J Magee 2015 [39]	cohort	Georgia	50		DM	any	318	89	229	9/9	Clinical	Phenotypic
Haiyan Zhang 2015 [40]	cross-sectional	China	NA		DM	primary	80	80	NA	4/11	Biochemical	Phenotypic
Xusheng Gao 2015 [41]	cross-sectional	China	54.0 ± 14.2		DM	secondary	207	50	157	7/11	Biochemical	Phenotypic
Jianhua Wei 2015 [42]	cross-sectional	China	NA		DM	any	190	80	110	5/11	Biochemical	Phenotypic
AD Salindri 2016 [43]	cohort	Georgia	49		DM	primary	268	36	232	9/9	Biochemical	Phenotypic
Zhihua Zhou 2016 [44]	cross-sectional	China	54.21 ± 14.3		DM	secondary	100	40	60	8/11	Biochemical	Phenotypic
CHI C LEUNG 2017 [45]	cohort	China (Hong Kong)	64.8 ± 13.6		DM	any	21414	3331	18083	9/9	Clinical	Phenotypic
M Muñoz-Torrico 2017 [46]	case-control	Mexico	49.5 ± 11.4		DM	any	90	49	41	8/9	Biochemical	Phenotypic
LM Perez-Navarro 2017 [47]	cohort	Mexico	50		T2DM	any	507	183	324	7/9	Biochemical	Phenotypic
Jielian Sun 2017 [48]	cohort	China	63.8 ± 12.4		DM	secondary	80	35	45	6/9	Clinical	Phenotypic
Chengling Luo 2017 [49]	case-control	China	59.79 ± 12.63		DM	any	294	58	236	7/9	Biochemical	Phenotypic
Hua Wang 2018 [50]	cohort	China	52.25 ± 12.39		DM	primary	296	126	170	7/9	Biochemical	Phenotypic
Ying Xi 2019 [51]	cross-sectional	China	NA		DM	any	499	235	264	8/11	Clinical	Phenotypic
M Lin 2020 [52]	cohort	China	NA		T2DM	any	732	207	525	6/9	Biochemical	Phenotypic
WM Song 2020 [53]	case-control	China	45–64		DM	secondary	1884	93	1791	9/9	Biochemical	Phenotypic
Qingya Wang 2020 [54]	cross-sectional	China	47.5–50.0		T2DM	any	1563	258	1305	8/11	Biochemical	Phenotypic
Qian Wu 2021 [55]	cross-sectional	China	56.55 ± 14.42		T2DM	any	936	76	860	10/11	Biochemical	Phenotypic
Weihua Hu 2021 [56]	cross-sectional	China	NA		T2DM	any	233	84	149	8/11	Biochemical	Phenotypic
Guihui Wu 2021 [57]	cross-sectional	China	55		DM	secondary	287	169	118	8/11	Biochemical	Phenotypic
Tao Guo 2021 [58]	cross-sectional	China	60.38 ± 11.88		T2DM	any	460	103	357	7/11	Biochemical	Phenotypic
GA Bermudez-Hernández 2022 [59]	case-control	Georgia, Mexico	45		T2DM	any	148	74	74	5/9	Clinical	Phenotypic
Lijuan Jian 2022 [60]	case-control	China	56.66 ± 12.89		DM	any	320	320	NA	5/9	Biochemical	Phenotypic
Wanmei Song 2022 [61]	case-control	China	48.76 ± 20.13		DM	any	9107	519	8588	6/9	Clinical	Phenotypic
Yingmei Zhang 2022 [62]	case-control	China	53		DM	any	357	141	216	8/9	Unclear	Both
Qiujing Chen 2022 [63]	cohort	China	58.26 ± 14.85		T2DM	any	267	267	NA	6/9	Clinical	Molecular
Yuanping Pan 2023 [64]	case-control	China	45		DM	any	513	186	327	6/9	Clinical	Phenotypic
GA Bermúdez-Hernández 2024 [65]	cohort	Mexico	56		T2DM	any	50	26	24	7/9	Clinical	Molecular
Xiaoxiao Cai 2024 [66]	cohort	China	45–59		T2DM	any	715	160	555	6/9	Clinical	Phenotypic
Yanting Yu 2024 [67]	case-control	China	47.65 ± 8.31		T2DM	any	204	102	102	7/9	Biochemical	Both
Nan Ni 2024 [68]	case-control	China	69.5 ± 6.5		T2DM	any	203	203	NA	7/9	Clinical	Phenotypic
Jing Guo 2024 [69]	case-control	China	47–66		T2DM	any	1422	541	881	7/9	Biochemical	Phenotypic
Ayinalem Alemu 2025 [70]	cross-sectional	Ethiopia	43.29		DM	any	76	76	NA	9/11	Biochemical	Both
Yalan Pi 2025 [71]	cross-sectional	China	57.55 ± 11.37		T2DM	any	111	55	56	10/11	Biochemical	Molecular

AHRQ – Agency for Healthcare Research and Quality, DM – diabetes mellitus, DR-TB – drug-resistant tuberculosis, DST – drug susceptibility testing, NA – not available, NOS – Newcastle-Ottawa Scale, TB – tuberculosis, T2DM – type 2 diabetes mellitus

*‘Any’ under DM type indicates unspecified or mixed diabetes type; ‘Any’ under DR-TB type indicates multiple or unspecified resistance patterns; ‘primary’ and ‘secondary’ refer to treatment-naïve and previously treated patients, respectively.

†Study quality was assessed using the Newcastle-Ottawa Scale for cohort and case-control studies (maximum score = 9) and the Agency for Healthcare Research and Quality tool for cross-sectional studies (maximum score = 11).

‡Biochemical ascertainment indicates diabetes defined using laboratory-based criteria; clinical ascertainment indicates diabetes defined using medical records, physician diagnosis, or self-report; unclear indicates that the ascertainment method was not clearly reported.

§Phenotypic indicates culture-based drug susceptibility testing; molecular indicates molecular assays; both indicates use of both phenotypic and molecular methods.

### Quality assessment

Most included studies were of high or moderate methodological quality. According to the predefined thresholds, two NOS-assessed studies were of low quality, whereas none of the AHRQ-assessed cross-sectional studies met the threshold for low quality; the remainder were of moderate or high quality. The main methodological shortcomings were limited control of confounding and incomplete reporting of exposure and outcome ascertainment. Specifically, among NOS-assessed cohort or case-control studies, Lingdi Lu (1999) and Xuguang Shao (2010) received low scores (≤4) because of limited sample representativeness, inadequate ascertainment of exposure and outcomes, and lack of adjustment for major confounders. Although Chunxiao Zhang (2008) and M. Lin (2020) met some selection criteria, they provided limited detail on comparability and outcome assessment, resulting in scores below the high-quality threshold. For cross-sectional studies assessed using AHRQ, Haiyan Zhang (2015) and Jianhua Wei (2015) scored only 4–5 of 11 items, with important limitations including unclear patient selection, inadequate handling of missing data, and ambiguous outcome definitions, thereby undermining reliability. In summary, the low-quality studies were characterised by weak representativeness, inadequate control of confounding, insufficient detail on outcome measurement, and incomplete methodological reporting (**Table 2**). Although retained in the primary analyses for completeness, sensitivity analyses excluding low-quality studies were also conducted to assess robustness.

**Table 2:** Risk-of-bias assessment of the included studies.

Panel A. Newcastle-Ottawa Scale-assessed cohort and case-control studies*																						
**Study author, year**		**Selection domain**								**Comparability domain**				**Outcome domain**						**Overall quality**		
		1		2		3		4		5				6		7		8				
Lingdi Lu 1999		*				*								*		*				Poor		
Mona Bashar 2001		*		*		*		*		**				*		*		*		Good		
Bachti Alisjahbana 2007		*		*		*				**				*		*		*		Good		
SP Fisher-Hoch 2008		*		*		*		*		**				*		*				Good		
Chunxiao Zhang 2008		*		*		*		*						*		*				Fair		
Xuguang Shao 2010		*		*		*								*						Poor		
Jenn-Tyang Chang 2011				*		*		*		**				*		*				Good		
Matthew J Magee 2013		*		*		*		*		*				*		*		*		Good		
Matthew J Magee 2015		*		*		*		*		**				*		*		*		Good		
AD Salindri 2016		*		*		*		*		**				*		*		*		Good		
Chi C Leung 2017		*		*		*		*		**				*		*		*		Good		
LM Perez-Navarro 2017		*		*		*		*		**						*		*		Good		
Jielian Sun 2017		*		*		*				*				*		*				Fair		
Chengling Luo 2017		*		*		*		*		**				*		*		*		Good		
M Muñoz-Torrico 2017		*		*		*		*		*				*		*				Good		
Hua Wang 2018		*		*		*				**				*		*				Good		
M Lin 2020		*		*		*				*				*				*		Fair		
WM Song 2020		*		*		*		*		**				*		*		*		Good		
GA Bermudez-Hernández 2022		*		*		*				*				*						Fair		
Lijuan Jian 2022		*		*		*				*				*						Fair		
Wanmei Song 2022		*		*		*				**				*						Fair		
Yingmei Zhang 2022		*		*		*		*		*				*		*		*		Good		
Qiujing Chen 2022		*		*		*		*		*				*						Fair		
Y Pan 2023		*		*		*		*		*				*						Fair		
GA Bermúdez-Hernández 2024		*		*		*		*		*				*		*				Good		
Xiaoxiao Cai 2024		*		*		*		*		*				*						Fair		
Yanting Yu 2024		*		*		*				*				*		*		*		Good		
Nan Ni 2024		*		*		*		*		*				*		*				Good		
Jing Guo 2024		*		*		*		*		*				*		*				Good		
**Panel B. Agency for Healthcare Research and Quality-assessed cross-sectional studies†**																					
**Study author, year**	**AHRQ items**																				**Overall score**
	1		2		3		4		5		6	7	8		9		10		11		
Huiping Duan 2012	yes		yes				yes				yes	yes					yes		NR		6
AH Hsu 2013	yes		yes				yes				yes	yes	yes				yes		yes		8
Yu Xiong 2013	yes		yes				yes					yes					yes		NR		5
Fengling Mi 2014	yes		yes		yes						yes	yes					yes		yes		7
Haiyan Zhang 2015	yes		yes		yes						yes	yes							NR		4
Xusheng Gao 2015	yes		yes		yes						yes	yes	yes				yes		NR		7
Jianhua Wei 2015	yes		yes								yes	yes							yes		5
Zhihua Zhou 2016	yes		yes		yes				yes		yes		yes		yes		yes		NR		8
Ying Xi 2019	yes		yes		yes				yes			yes	yes				yes		yes		8
Qingya Wang 2020	yes		yes				yes		yes			yes			yes		yes		yes		8
Q Wu 2021	yes		yes		yes		yes		yes		yes	yes	yes				yes		yes		10
Weihua Hu 2021	yes		yes		yes		yes				yes	yes	yes		yes				NR		8
Guihui Wu 2021	yes		yes		yes		yes				yes		yes		yes				yes		8
Tao Guo 2021	yes		yes				yes		yes		yes		yes						NR		7
A Alemu 2025	yes		yes		yes		yes		yes		yes		yes				yes		yes		9
Yalan Pi 2025	yes		yes		yes		yes		yes			yes	yes		yes		yes		yes		10

AHRQ – Agency for Healthcare Research and Quality, NOS – Newcastle-Ottawa Scale

*Indicates that one star was awarded for the item, ** indicates that two stars were awarded in the comparability domain, and a blank cell indicates that no star was awarded because the item was not fulfilled or was not clearly reported. Overall quality was classified as good, fair, or poor according to the total NOS score.

†For AHRQ-assessed studies, ‘yes’ indicates that the item was fulfilled, a blank cell indicates that the item was not fulfilled or was not clearly reported, and ‘NR’ indicates not reported. Overall scores of 8–11 were considered good, 4–7 fair, and 0–3 poor.

### Drug resistance rates

Overall, the pooled prevalence of drug resistance was generally higher among TB patients with DM than among those without. For example, the prevalence of MDR-TB was 2% (95% CI = 1.9–2.0%) in the DM group *vs*. 1% (95% CI = 1.0–1.1%) in the non-DM group. The narrow confidence intervals for these prevalence estimates reflect the large sample sizes contributing to the analyses, but the high heterogeneity (*I*^2^>80% for MDR-TB) indicates substantial variability across studies, likely due to differences in study populations, resistance patterns, and background epidemiology. Therefore, these pooled prevalence estimates should be interpreted as averages that mask considerable variation between studies. Detailed prevalence estimates and forest plots are provided in Figures S2–6 in the **Online Supplementary Document**. According to the predefined thresholds, two studies assessed using NOS were rated as low quality, whereas none of the cross-sectional studies assessed using the AHRQ tool were rated as low quality.

#### Primary analysis

**Figure 2:**
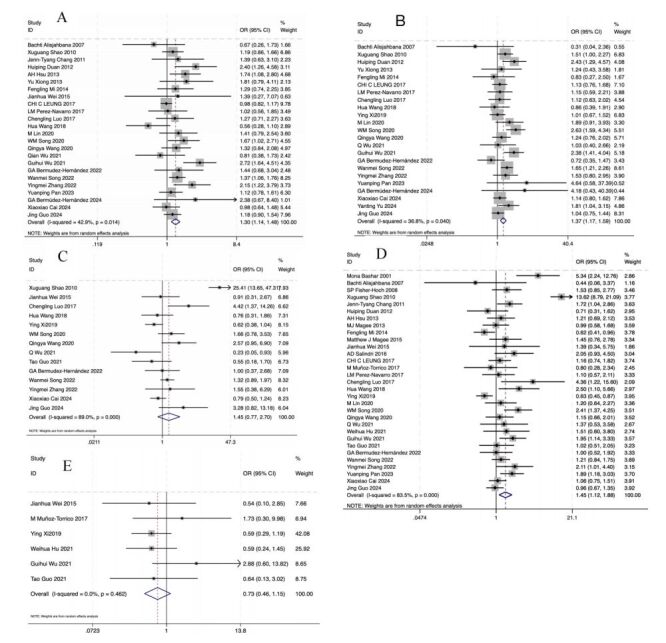
Associations between diabetes mellitus and different categories of drug-resistant tuberculosis. **Panel A**. Isoniazid-resistant tuberculosis. **Panel B**. Rifampicin-resistant tuberculosis. **Panel C**. Polydrug-resistant tuberculosis. **Panel D**. Multidrug-resistant tuberculosis. **Panel E**. Extensively drug-resistant tuberculosis. In each forest plot, the square represents the study-specific effect estimate, the horizontal line indicates the corresponding 95% CI, and the diamond represents the pooled estimate. CI – confidence interval, DM – diabetes mellitus, DR-TB – drug-resistant tuberculosis, HR-TB – isoniazid-resistant tuberculosis, MDR-TB – multidrug-resistant tuberculosis, OR – odds ratio, PDR-TB – polydrug-resistant tuberculosis, RR-TB – rifampicin-resistant tuberculosis, XDR-TB – extensively drug-resistant tuberculosis.

Meta-analysis showed that DM was associated with higher odds of isoniazid-resistant TB (OR = 1.30; 95% CI = 1.14–1.48, *I*^2^ = 42.9%; 95% prediction interval (PI) = 0.98–1.72) and rifampicin-resistant TB (OR = 1.37; 95% CI = 1.17–1.59; *I*^2^ = 36.8%; 95% PI = 1.02–1.84). The pooled association for MDR-TB was also positive (OR = 1.45; 95% CI = 1.12–1.88, *I*^2^ = 83.5%; 95% PI = 0.72–2.92), although heterogeneity was substantial. Associations for polydrug-resistant TB (OR = 1.45; 95% CI = 0.77–2.70, *I*^2^ = 89.0%) and extensively drug-resistant TB (OR = 0.73; 95% CI = 0.46–1.15, *I*^2^ = 0%) were imprecise and were based on relatively few studies (**Figure 2**).

The number of contributing studies varied across resistance categories. Analyses of isoniazid-resistant TB (n = 23), rifampicin-resistant TB (n = 23), and MDR-TB (n = 30) were based on larger evidence sets, whereas analyses of polydrug-resistant TB (n = 12) and extensively drug-resistant TB (n = 6) relied on smaller subsets of studies and should therefore be interpreted more cautiously.

#### Subgroup analysis

Selected subgroup analyses are summarised in Table 3. For MDR-TB, the pooled association appeared stronger in longitudinal studies (OR = 1.66; 95% CI = 1.16–2.38) than in cross-sectional studies (OR = 1.01; 95% CI = 0.75–1.36). Analyses stratified by resistance history also suggested elevated odds in studies focused on primary MDR-TB (OR = 2.25; 95% CI = 1.28–3.97) and secondary MDR-TB (OR = 2.16; 95% CI = 1.46–3.18), although these estimates were derived from relatively few studies and should be interpreted with caution.

**Table 3:** Selected subgroup analyses of the association between DM and drug-resistant tuberculosis

Factor	Subgroup	DR-TB type	No. of studies	Pooled OR (95% CI)*	*I*^2^ (%)	*P* heterogeneity†
Study design	Cross-sectional	HR-TB	7	1.65 (1.24–2.19)	41.0	0.118
		RR-TB	7	1.41 (1.02–1.97)	44.5	0.094
		PDR-TB	4	0.72 (0.31–1.67)	68.2	0.024
		MDR-TB	10	1.01 (0.75–1.36)	54.8	0.018
	Longitudinal	HR-TB	16	1.18 (1.04–1.34)	27.6	0.146
		RR-TB	16	1.35 (1.13–1.61)	37.3	0.066
		PDR-TB	8	2.36 (0.95–5.86)	92.1	<0.001
		MDR-TB	18	1.66 (1.16–2.38)	86.4	<0.001
Resistance history	Primary MDR-TB	HR-TB	2	0.98 (0.31–3.09)	78.4	0.031
		RR-TB	2	0.98 (0.52–1.86)	0.0	0.587
		MDR-TB	2	2.25 (1.28–3.97)	0.0	0.734
	Secondary MDR-TB	HR-TB	2	2.12 (1.31–3.42)	46.5	0.172
		RR-TB	2	2.51 (1.74–3.61)	0.0	0.793
		MDR-TB	2	2.16 (1.46–3.18)	0.0	0.590
Geographic region	All regions	HR-TB	20	1.23 (1.10–1.37)	17.1	0.241
		RR-TB	20	1.33 (1.15–1.54)	28.8	0.112
		PDR-TB	12	1.51 (0.73–3.11)	90.5	<0.001
		MDR-TB	27	1.37 (1.03–1.82)	84.8	<0.001
	East Asia	HR-TB	19	1.32 (1.14–1.52)	51.5	0.005
		RR-TB	19	1.41 (1.21–1.65)	36.7	0.056
		PDR-TB	12	1.37 (0.68–2.73)	90.3	<0.001
		MDR-TB	22	1.49 (1.08–2.05)	87.3	<0.001
	Latin America	MDR-TB	4	2.10 (1.21–3.65)	51.2	0.103
	USA/Europe	MDR-TB	5	1.70 (1.12–2.58)	48.5	0.099
	Other	MDR-TB	3	1.30 (0.67–2.52)	55.3	0.105
DM type	T2DM	HR-TB	11	1.17 (1.00–1.37)	0.0	0.844
		RR-TB	11	1.16 (0.98–1.38)	0.0	0.470
		PDR-TB	5	0.80 (0.43–1.47)	47.6	0.106
		MDR-TB	13	1.07 (0.91–1.26)	8.3	0.363
	DM, unspecified	HR-TB	13	1.41 (1.15–1.74)	64.9	0.001
		RR-TB	12	1.53 (1.24–1.89)	44.8	0.046
		PDR-TB	7	2.38 (0.80–7.07)	93.8	<0.001
		MDR-TB	18	1.78 (1.17–2.70)	88.8	<0.001
DM ascertainment	Biochemical criteria	MDR-TB	20	1.38 (1.08–1.76)	65.3	–
	Clinical/self-report	MDR-TB	5	1.58 (0.95–2.63)	89.1	–
DST method	Phenotypic	MDR-TB	28	1.42 (1.10–1.84)	84.5	–
	Molecular	MDR-TB	2	1.51 (0.91–2.51)	0.0	–

CI – confidence interval, DM – diabetes mellitus, DR-TB – drug-resistant tuberculosis, DST – drug susceptibility testing, HR-TB – isoniazid-resistant tuberculosis, MDR-TB – multidrug-resistant tuberculosis, OR – odds ratio, PDR-TB – polydrug-resistant tuberculosis, RR-TB – rifampicin-resistant tuberculosis, T2DM – type 2 diabetes mellitus, XDR-TB – extensively drug-resistant tuberculosis.

*Summary of pooled odds ratios and 95% confidence intervals for subgroups defined by study design, resistance history, geographic region, diabetes type, diabetes ascertainment method, and drug susceptibility testing method.

†*P* heterogeneity refers to the Cochran Q test within each subgroup. An en dash indicates that the test was not reported or was not applicable because of the small number of studies.

For MDR-TB, studies using standardised biochemical criteria for diabetes ascertainment, such as fasting blood glucose, glycated haemoglobin, or oral glucose tolerance testing, yielded a pooled OR = 1.38 (95% CI = 1.08–1.76, *I*^2^ = 65.3%), whereas studies relying on clinical records or self-report yielded an OR = 1.58 (95% CI = 0.95–2.63, *I*^2^ = 89.1%). Directionally similar results were observed in studies using phenotypic drug susceptibility testing (OR = 1.42; 95% CI = 1.10–1.84) and molecular drug susceptibility testing (OR = 1.51; 95% CI = 0.91–2.51), although the molecular subgroup was based on relatively few studies.

Geographic subgroup analyses suggested possible regional variability. The association between DM and MDR-TB appeared strongest in studies from Latin America (OR = 2.10; 95% CI = 1.21–3.65) and from the USA/Europe (OR = 1.70; 95% CI = 1.12–2.58). In studies from East Asia, the pooled estimate remained positive (OR = 1.49; 95% CI = 1.08–2.05, *I*^2^ = 87.3%), although heterogeneity was substantial. These regional findings should be interpreted cautiously because the evidence base was highly unbalanced, with most included studies originating from East Asia and relatively few studies available from other regions. Differences between studies restricted to type 2 diabetes mellitus (T2DM) and those with unspecified diabetes type were modest and were more consistent with variation in study design and reporting than with clearly distinct biological effects (**Table 3**).

Time-period analyses showed broadly stable estimates over time, with no statistically significant temporal trend (*P* = 0.42) (Table S1 in the **Online Supplementary Document**).

### Impact of glycaemic control on drug resistance

In this exploratory, hypothesis-generating analysis, we examined the association between glycaemic control and any drug-resistant TB among studies reporting glycated haemoglobin (HbA1c) data. Because only a limited number of studies were available (n = 8) and study-specific HbA1c thresholds were not fully uniform, these findings should be interpreted with caution. A pooled analysis comparing patients with poorer glycaemic control (HbA1c ≥7.0%) and those with better glycaemic control (HbA1c <7.0%) showed that poorer control was associated with higher odds of any drug-resistant TB (OR = 2.05; 95% CI = 1.25–3.36, *I*^2^ = 25.0%). Although these findings are exploratory and hypothesis-generating, they support the need for more standardised reporting of HbA1c in future studies to enable more robust analyses of the relationship between glycaemic control and drug resistance. This should not be interpreted as evidence of a confirmed dose-response relationship. Detailed results are presented in Figure S7 in the **Online Supplementary Document**.

### Meta-regression and heterogeneity

To explore potential sources of heterogeneity among studies, meta-regression analyses were conducted for sample size, study region, drug resistance type, and DM type classification. None of these variables, including sample size (*P* = 0.17), study region (*P* = 0.58), drug resistance type (*P* = 0.26), or DM type (*P* = 0.41), showed a statistically significant association with heterogeneity (all *P* > 0.05). The absence of statistically significant findings may be due to limited statistical power, as well as the use of study-level rather than individual-level covariates. Overall, these results suggest that the observed heterogeneity is more likely attributable to unmeasured confounding factors or underlying clinical differences between studies.

Key potential confounders that were not consistently reported or adjusted for across the included studies include human immunodeficiency virus (HIV) status, socioeconomic status (*e.g.* income, education, living conditions), smoking, alcohol consumption, and nutritional status (*e.g*. body mass index). For instance, HIV co-infection is a powerful risk factor for both TB and the acquisition of drug resistance, and its differential distribution across study populations could significantly influence the observed DM-DR-TB association. Similarly, poor nutrition can impair immune function and affect drug metabolism, potentially acting as a strong confounder or effect modifier. The inability to control for these factors in our aggregate data meta-analysis represents a significant limitation.

### Publication bias

**Figure 3:**
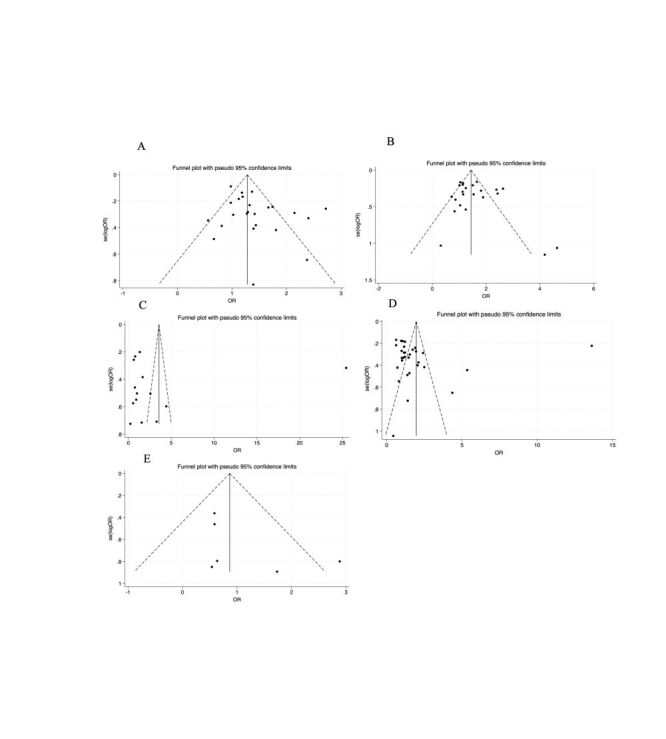
Funnel plots assessing potential publication bias in the association between diabetes mellitus and drug-resistant tuberculosis. **Panel A**. Isoniazid-resistant tuberculosis. **Panel B**. Rifampicin-resistant tuberculosis. **Panel C**. Polydrug-resistant tuberculosis. **Panel D**. Multidrug-resistant tuberculosis. **Panel E**. Extensively drug-resistant tuberculosis. The horizontal axis shows the log OR, and the vertical axis shows the standard error. The vertical line indicates the pooled effect estimate, and the diagonal dashed lines indicate pseudo 95% confidence limits. CI – confidence interval, DM – diabetes mellitus, DR-TB – drug-resistant tuberculosis, HR-TB – isoniazid-resistant tuberculosis, MDR-TB – multidrug-resistant tuberculosis, OR – odds ratio, PDR-TB – polydrug-resistant tuberculosis, RR-TB – rifampicin-resistant tuberculosis, SE – standard error, XDR-TB – extensively drug-resistant tuberculosis.

Publication bias was assessed using funnel plots and Egger’s test. The funnel plot appeared symmetric, suggesting a low likelihood of bias (**Figure 3**). Egger’s and Begg’s tests were consistent with this observation (*P* > 0.10 for all outcomes). Application of the trim-and-fill method produced effect estimates that remained consistent with the original pooled results, further supporting robustness. However, given the high heterogeneity observed for certain outcomes (*e.g.* MDR-TB), the statistical power of Egger’s test may be limited. Therefore, Supplementary Doi plot analyses (Figure S1 in the **Online Supplementary Document**) suggested minor asymmetry for isoniazid-resistant TB, rifampicin-resistant TB, and multidrug-resistant TB, no asymmetry for polydrug-resistant TB, and major asymmetry for extensively drug-resistant TB; however, these assessments should be interpreted cautiously given the small number of studies for some outcomes. It should also be acknowledged that the inclusion of studies published in Chinese journals may introduce potential language or regional publication bias, although sensitivity analyses excluding these studies did not materially alter the main findings.

### Sensitivity analysis

Leave-one-out sensitivity analyses confirmed the stability of pooled estimates. Furthermore, sensitivity analyses using the Hartung-Knapp-Sidik-Jonkman method [26] for MDR-TB and PDR-TB produced pooled estimates consistent with the main random-effects model, although with wider confidence intervals, reflecting the high heterogeneity and providing a more conservative estimate of uncertainty.

## DISCUSSION

### Principal findings

In this systematic review and meta-analysis of 45 studies involving 51 982 participants, DM was associated with higher odds of HR-TB, RR-TB, and MDR-TB. By contrast, the associations with PDR-TB and extensively drug-resistant TB were imprecise and were based on relatively small numbers of studies. These findings should be interpreted as associations rather than evidence of causality because all included studies were observational. Nevertheless, the results are broadly consistent with previous meta-analyses reporting increased odds of MDR-TB among patients with TB and DM [9-12]. Unlike earlier evidence syntheses that focused mainly on MDR-TB, the present review examined multiple resistance categories and suggests that DM may be associated not only with more advanced resistance phenotypes, but also with earlier resistance events such as isoniazid resistance [15].

### Interpretation in relation to existing evidence

The observed associations are biologically and clinically plausible. Hyperglycaemia may impair host defence by reducing macrophage and neutrophil phagocytic activity, limiting chemotaxis, and impairing T-cell-mediated immunity, thereby facilitating the persistence of *Mycobacterium tuberculosis (MTB)* [5,13]. Pharmacological mechanisms may also contribute. A recent systematic review reported that DM is associated with altered pharmacokinetics of several anti-TB drugs, with rifampicin exposure being consistently lower in patients with type 2 DM [7]. Earlier pharmacokinetic studies similarly suggested reduced exposure to rifampicin and other anti-TB agents in this context [8,72]. Together, these immunological and pharmacological pathways provide a plausible explanation for the higher odds of drug-resistant TB observed in patients with DM.

The finding for isoniazid-resistant TB is particularly noteworthy. Isoniazid resistance may represent an early stage in the pathway towards more complex resistance phenotypes [15]. The positive association observed in the present analysis therefore raises the possibility that DM may influence resistance development earlier than has often been recognised. This interpretation remains tentative, but it is consistent with the broader hypothesis that metabolic dysfunction may contribute to both impaired bacillary clearance and suboptimal drug exposure.

By contrast, the null finding for extensively drug-resistant TB should be interpreted cautiously. This result may simply reflect limited statistical power, as relatively few studies contributed to that analysis. It is also possible that the influence of DM is more readily detectable for resistance involving first-line drugs than for more complex resistance patterns that accumulate over time through multiple biological, treatment-related, and programmatic pathways. Larger longitudinal studies will be needed to clarify whether DM is also associated with extensively drug-resistant TB. The importance of this question is underscored by recent evidence showing poor outcomes and high mortality during treatment for drug-resistant TB among individuals with DM, particularly those with low body mass index [73].

### Subgroup analyses and potential effect modifiers

The subgroup analyses provide additional context, although they should be interpreted cautiously. For MDR-TB, the pooled association appeared stronger in longitudinal studies (OR = 1.66; 95% CI = 1.16–2.38) than in cross-sectional studies (OR = 1.01; 95% CI = 0.75–1.36). While this pattern is consistent with a temporal relationship in which DM precedes drug resistance, it does not establish causality, because residual confounding and differential case mix cannot be excluded. Similarly, elevated odds were observed in studies addressing both primary and secondary MDR-TB, suggesting that DM may be associated with both de novo resistance and resistance emerging in the context of previous treatment. However, these subgroup estimates were based on relatively small numbers of studies and should not be overinterpreted (**Table 3**).

Geographic subgroup analyses also suggested possible regional variation. The pooled association between DM and MDR-TB appeared stronger in Latin America and in the USA/Europe than in East Asia. However, the evidence base was heavily dominated by studies from East Asia, especially China, whereas the non-East Asian strata were based on relatively few studies. The apparent regional differences may therefore reflect not only true epidemiological variation, but also differences in study design, baseline resistance prevalence, access to drug susceptibility testing, treatment programmes, and local distributions of Mycobacterium tuberculosis lineages. These findings should therefore be viewed as hypothesis-generating rather than definitive (**Table 3**).

The exploratory analysis of glycaemic control suggests that poorer control may amplify the risk of drug-resistant TB. Patients with poorer glycaemic control had higher odds of drug resistance than those with better control (OR = 2.05; 95% CI = 1.25–3.36), which is consistent with previous literature linking poor glycaemic control to less favourable TB outcomes [6,16]. However, this finding should not be interpreted as a confirmed dose-response relationship. Only a limited number of studies reported glycaemic data, and thresholds for glycated haemoglobin varied across studies. More standardised reporting will be required before firmer conclusions can be drawn.

### Clinical and public health implications

From a clinical and public health perspective, these findings support closer integration of TB and DM care. Patients with TB and comorbid DM may benefit from more systematic assessment of glycaemic status and careful consideration of drug susceptibility testing, particularly in settings with a high background prevalence of drug-resistant TB. However, because the present review is based on observational evidence, the findings do not by themselves justify universal changes in diagnostic policy. Rather, they support the view that DM should be considered an important factor in risk stratification and clinical decision-making.

The results also suggest that strengthening bidirectional screening and integrated care models may be beneficial. Such approaches could combine routine assessment for DM in patients with TB, improved glycaemic monitoring during TB treatment, timely access to drug susceptibility testing, and adherence support. Although the exploratory glycaemic control analysis raises the possibility that improved glycaemic management might reduce the risk of drug resistance, this hypothesis should be tested in prospective and interventional studies before firm clinical recommendations are made. These considerations are consistent with recent calls for more coordinated TB-DM strategies in high-burden settings [74–77].

### Strengths and limitations

This review has several strengths. To our knowledge, it is among the first evidence syntheses to evaluate multiple categories of TB drug resistance in relation to DM rather than focusing solely on MDR-TB. It also included a relatively large pooled sample and incorporated subgroup, sensitivity, and supplementary analyses to explore heterogeneity and robustness. In addition, the review included both international and Chinese databases, which increased the likelihood of identifying relevant studies from settings with high burdens of both TB and DM.

Several limitations should also be acknowledged. First, substantial heterogeneity was observed for some outcomes, particularly MDR-TB (*I*^2^ = 83.5%). This heterogeneity was not explained by the meta-regression variables examined and likely reflects a combination of differences in local epidemiology, prior treatment exposure, laboratory methods, case mix, and unmeasured patient-level factors. Second, because many included studies were cross-sectional, causal inference is limited. Even in longitudinal studies, residual confounding remains a major concern. Important factors such as HIV status, smoking, alcohol use, nutritional status, socioeconomic conditions, and prior TB treatment were not consistently measured or adjusted for across the primary studies. Third, the evidence base was geographically unbalanced, with most studies originating from East Asia, particularly China. As a result, the pooled estimates may be most applicable to East Asian settings, and extrapolation to other regions, especially those with higher burdens of TB/HIV co-infection or different drug-resistant TB strain profiles, should be undertaken cautiously. Fourth, inconsistent reporting of glycaemic control, including variation in glycated haemoglobin thresholds and measurement approaches, limited more refined analyses of effect modification. Finally, no included studies specifically focused on type 1 DM, so no conclusions can be drawn for that subgroup.

### Overall interpretation

In summary, this meta-analysis indicates that DM is associated with higher odds of isoniazid-resistant TB, rifampicin-resistant TB, and MDR-TB. The available evidence also suggests that poorer glycaemic control may be associated with higher odds of drug-resistant TB, although this finding remains exploratory. Taken together, these results support the importance of integrated approaches to TB and DM care and highlight the need for future research using prospective designs, standardised definitions of glycaemic status, and more comprehensive adjustment for key confounders to clarify the mechanisms linking DM and drug-resistant TB.

## CONCLUSIONS

In conclusion, DM was associated with higher odds of isoniazid-resistant tuberculosis, rifampicin-resistant tuberculosis, and multidrug-resistant tuberculosis. By contrast, the evidence for polydrug-resistant tuberculosis and extensively drug-resistant tuberculosis remained limited and imprecise. These findings should be interpreted as associations rather than evidence of causality, particularly in view of the substantial between-study heterogeneity, the possibility of residual confounding, and the geographic concentration of the available evidence in East Asia. Overall, the results support closer integration of tuberculosis and diabetes assessment in clinical care and public health practice. However, the extent to which earlier drug susceptibility testing strategies or specific glycaemic targets can improve outcomes should be clarified in future prospective multicentre studies.

## Additional material


Online Supplementary Document
