# Supplementary material for: Impact of diabetes mellitus on drug-resistant tuberculosis across resistance categories: a systematic review and meta-analysis
Source: J Glob Health. 2026 May 12;16:04170. doi: 10.7189/jogh.16.04170 (PMC13261747; doi:10.7189/jogh.16.04170)
Supplement: Online Supplementary Document [file jogh-16-04170-s001.pdf]

Supplement to: Gou B, Wang J, Liu H, Lu S, Wang X. Impact of diabetes mellitus on drug-resistant tuberculosis across resistance categories: a systematic review and meta-analysis. J Glob Health. 2026;16:04170.

Impact of diabetes mellitus on drug-resistant tuberculosis across resistance categories: a systematic review and meta-analysis

Boya Gou[1,2], Jin Wang[1], Han Liu[1], Shuihua Lu[1*], Xiaomin Wang[1*]

[1]National Clinical Research Center for Infectious Diseases, Shenzhen Third People's Hospital, Shenzhen 518112, China.

[2]Department of Internal Medicine, Shenzhen University Health Science Center, Shenzhen University, Shenzhen 518060, China.Boya Gou, Jin Wang, Han Liu, Shuihua Lu, Xiaomin Wang

Correspondence: Xiaomin Wang (WXM_ZMU@163.com); Shuihua Lu (lushuihua66@126.com)

Supplementary Material

Supplementary Material 1: Search strategies. 1

Supplementary Table

Supplementary Table S1: Temporal trends in the association between diabetes mellitus and MDR-TB...... 3

Supplementary Figures

Supplementary Figure S1: Doi plots 4

Supplementary Figure S2-S6: Forest plots of prevalence estimates.. 6

Supplementary Figure S7 Forest plot of DR-TB by glycaemic control... 11

19

Supplementary Material

Supplementary Material 1: Search strategies. .

A total of 2098 records were identified across seven databases.

PubMed (n = 378)

(("tuberculosis" OR "pulmonary tuberculosis" OR "extrapulmonary tuberculosis" OR "lung tuberculosis" OR "Mycobacterium tuberculosis" OR "M. tuberculosis" OR "MTB" OR "Mycobacterium bovis" OR "Mycobacterium tuberculosis"[Mesh] OR "Mycobacterium bovis"[Mesh] OR "Tuberculosis"[Mesh] OR "Tuberculosis, Pulmonary"[Mesh] OR "Tuberculosis, Extrapulmonary"[Mesh]) AND ("drug resistance" OR "drug resistant" OR "isoniazid resistant" OR "rifampicin resistant" OR "multidrug resistant" OR "MDR" OR "XDR" OR "Drug Resistance, Bacterial"[Mesh]) AND ("diabetes" OR "diabetes mellitus" OR "type 1 diabetes mellitus" OR "type 2 diabetes mellitus" OR "Diabetes Mellitus"[Mesh] OR "Diabetes Mellitus, Type 1"[Mesh] OR "Diabetes Mellitus, Type 2"[Mesh]))

Scopus (n = 759)

(("tuberculosis" OR "pulmonary tuberculosis" OR "extrapulmonary tuberculosis" OR "Mycobacterium tuberculosis" OR "M. tuberculosis" OR "MTB" OR "Mycobacterium bovis" OR "TB") AND ("drug resistance" OR "drug resistant" OR "isoniazid resistant" OR "rifampicin resistant" OR "multidrug resistant" OR "MDR" OR "XDR") AND ("diabetes" OR "diabetes mellitus" OR "type 1 diabetes" OR "type 2 diabetes"))

ScienceDirect (n = 64)

("tuberculosis" OR "Mycobacterium tuberculosis" OR "TB" OR "MTB") AND ("drug resistance" OR "multidrug resistant" OR "MDR" OR "XDR" OR "isoniazid resistant" OR "rifampicin resistant") AND ("diabetes" OR "diabetes mellitus" OR "type 1 diabetes" OR "type 2 diabetes")

Web of Science (n = 209)

TS=(("tuberculosis" OR "pulmonary tuberculosis" OR "extrapulmonary tuberculosis" OR "Mycobacterium tuberculosis" OR "M. tuberculosis" OR "MTB" OR "Mycobacterium bovis" OR "TB") AND TS=("drug resistance" OR "drug resistant" OR "isoniazid resistant" OR "rifampicin resistant" OR "multidrug resistant" OR "MDR" OR "XDR") AND TS=("diabetes" OR "diabetes mellitus" OR "T1DM" OR "T2DM"))

Wanfang Data (n = 266)

主题: (("结核" OR "肺结核" OR "肺外结核" OR "结核病" OR "结核分枝杆菌" OR "结核性脑膜炎" OR "TB" OR "MTB") AND 主题=("耐药" OR "药物耐药" OR "异烟肼耐药" OR "利福平耐药" OR "多药耐药" OR "广泛耐药" OR "MDR" OR "XDR") AND 主题=("糖尿病" OR "1 型糖尿病" OR "2 型糖尿病"))

VIP (n = 97)

M=((结核 OR 肺结核 OR 肺外结核 OR 结核病 OR 结核性脑膜炎 OR 结核分枝杆菌 OR TB OR MTB) AND M=(耐药 OR 多药耐药 OR 广泛耐药 OR 异烟肼耐药 OR 利福平耐药 OR MDR OR XDR) AND M=(糖尿病 OR 1 型糖尿病 OR 2 型糖尿病))

CNKI (n = 325)

SU=(("结核" OR "肺结核" OR "肺外结核" OR "结核病" OR "结核分枝杆菌" OR "结核性脑膜炎" OR "TB" OR "MTB") AND SU=("耐药" OR "多药耐药" OR "广泛耐药" OR "异烟肼耐药" OR "利福平耐药" OR "MDR" OR "XDR") AND SU=("糖尿病" OR "1 型糖尿病" OR "2 型糖尿病"))

Supplementary Table

Table S1. Temporal trends in the association between diabetes mellitus and multidrug-resistant tuberculosis.

Time Period	Number of Studies	Pooled OR (95% CI)	I^2 (%)	P for trend
Pre-2010	5	1.51 (1.08, 2.11)	71.2	Reference
2010 – 2015	8	1.42 (1.02, 1.98)	68.5	
2016 – 2020	10	1.48 (1.18, 1.86)	65.9	
Post-2020	7	1.39 (1.05, 1.84)	70.1	0.42

CI – confidence interval, MDR-TB – multidrug-resistant tuberculosis, OR – odds ratio.

Supplementary Figure

Figure S1. Doi plots.

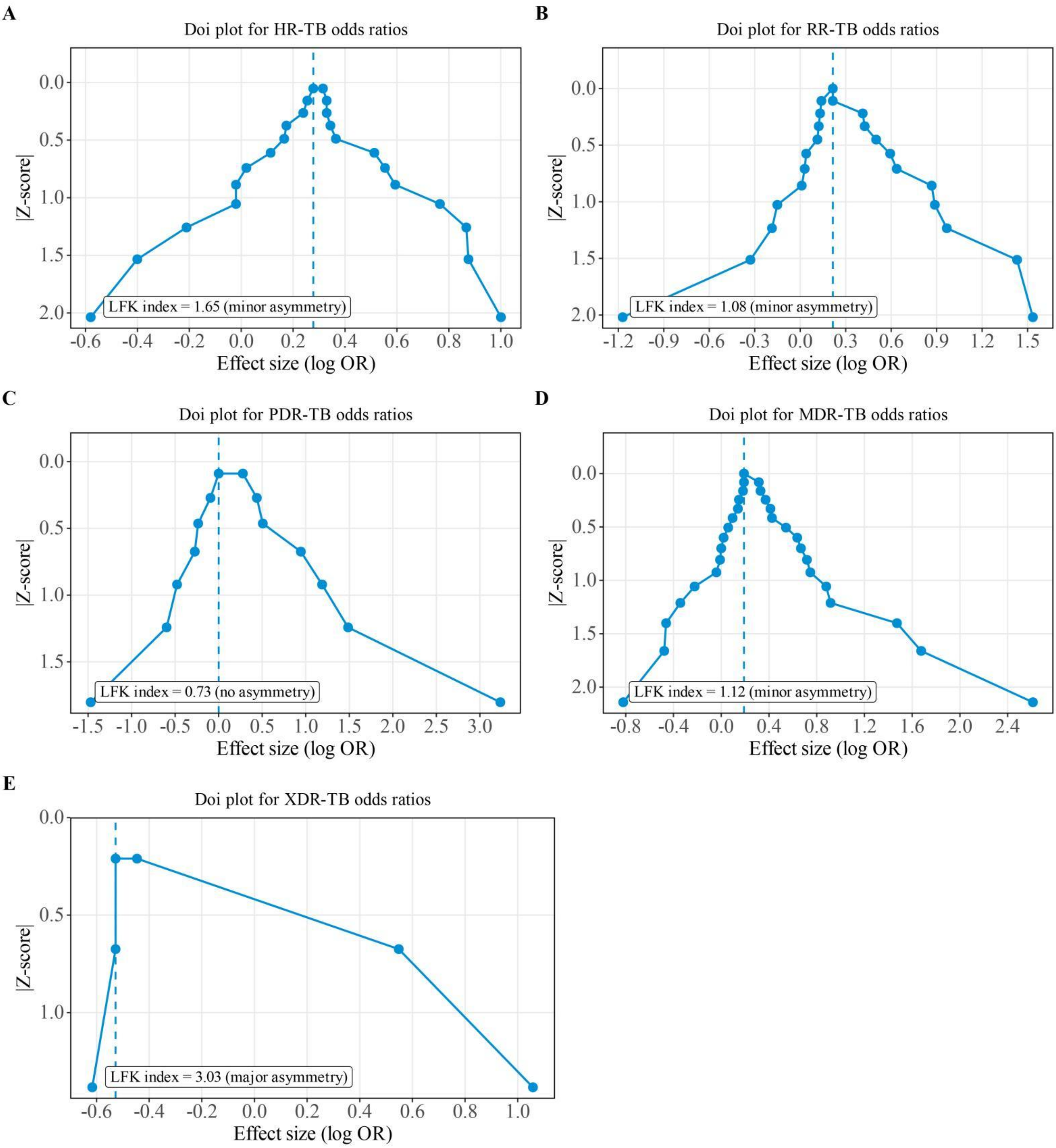

Figure S1. Doi plots assessing small-study effects for the associations between diabetes mellitus and different drug-resistant tuberculosis outcomes. Panel A. Isoniazid-resistant tuberculosis. Panel B. Rifampicin-resistant tuberculosis. Panel C. Polydrug-resistant tuberculosis. Panel D. Multidrug-resistant tuberculosis. Panel E. Extensively drug-resistant tuberculosis. The horizontal axis shows the effect size (log odds ratio), and the vertical axis shows the absolute Z-score. The vertical dashed line indicates the reference position in each Doi plot. The Luis

Furuya-Kanamori index values were 1.65 for isoniazid-resistant tuberculosis, 1.09 for rifampicin-resistant tuberculosis, 0.73 for polydrug-resistant tuberculosis, 1.13 for multidrug-resistant tuberculosis, and 3.03 for extensively drug-resistant tuberculosis, indicating minor asymmetry for isoniazid-resistant tuberculosis, rifampicin-resistant tuberculosis, and multidrug-resistant tuberculosis, no asymmetry for polydrug-resistant tuberculosis, and major asymmetry for extensively drug-resistant tuberculosis. HR-TB – isoniazid-resistant tuberculosis, LFK – Luis Furuya-Kanamori, MDR-TB – multidrug-resistant tuberculosis, OR – odds ratio, PDR-TB – polydrug-resistant tuberculosis, RR-TB – rifampicin-resistant tuberculosis, XDR-TB – extensively drug-resistant tuberculosis.

Figure S2-S6

Supplementary prevalence forest plots

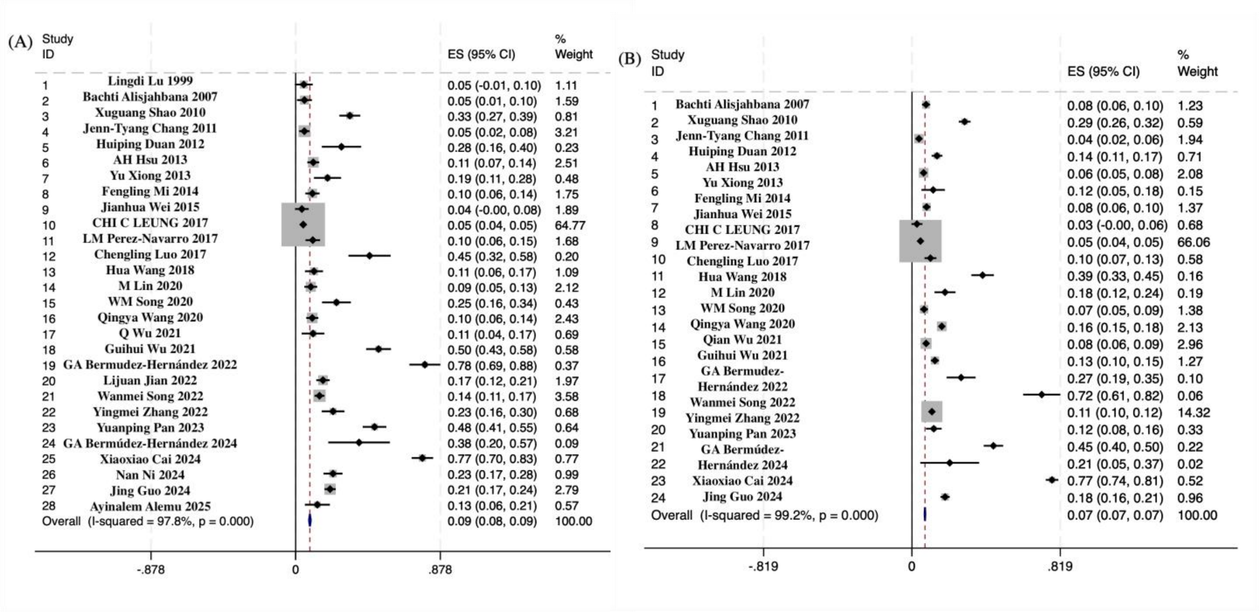

Figure S2. Prevalence of isoniazid-resistant tuberculosis among patients with diabetes mellitus and among tuberculosis patients overall. Panel A. Prevalence among individuals with diabetes mellitus. Panel B. Prevalence among tuberculosis patients overall. CI – confidence interval, DM – diabetes mellitus, ES – effect size, HR-TB – isoniazid-resistant tuberculosis, TB – tuberculosis.

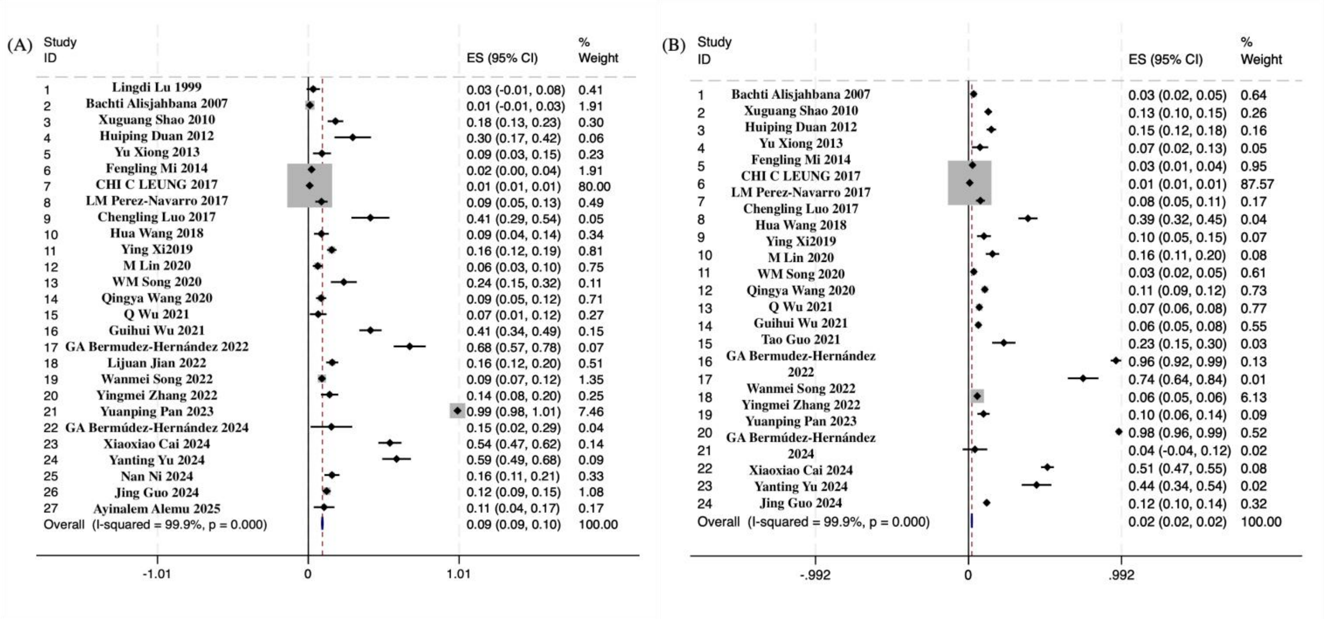

Figure S3. Prevalence of rifampicin-resistant tuberculosis among patients with diabetes mellitus and among tuberculosis patients overall. Panel A. Prevalence among individuals with diabetes mellitus. Panel B. Prevalence among tuberculosis patients overall. CI – confidence interval, DM – diabetes mellitus, ES – effect size, RR-TB – rifampicin-resistant tuberculosis, TB – tuberculosis.

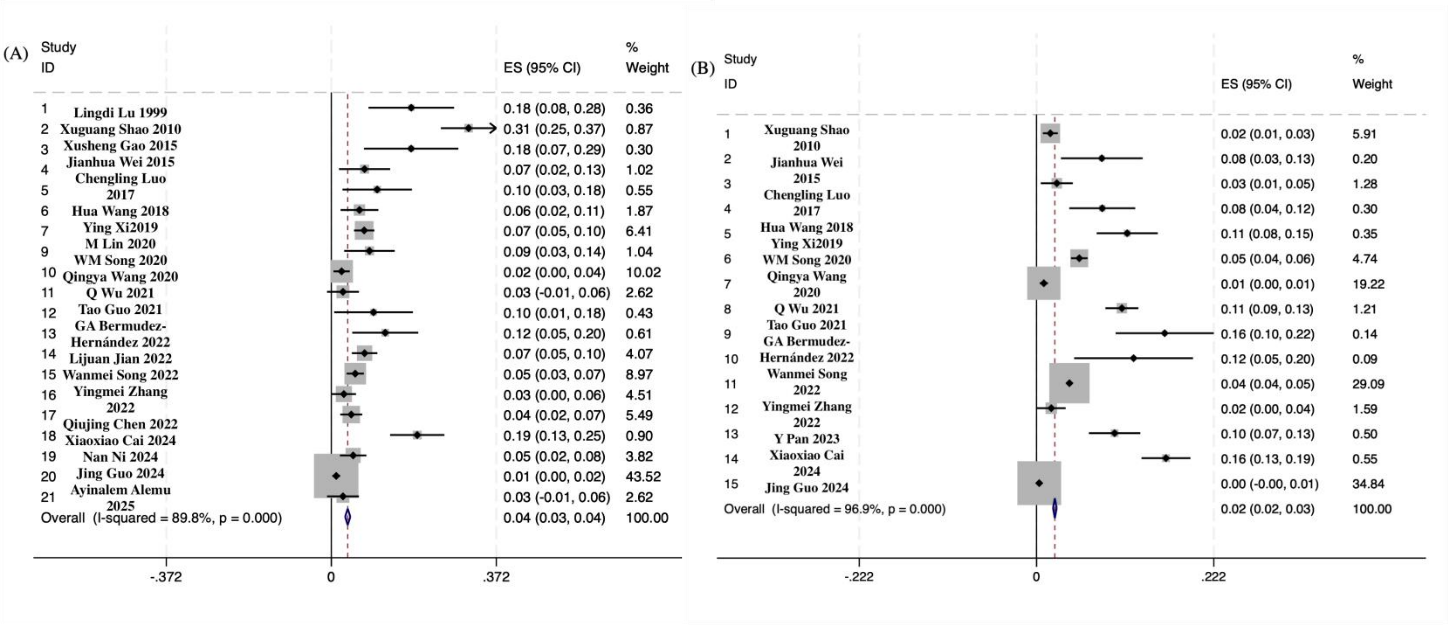

Figure S4. Prevalence of polydrug-resistant tuberculosis among patients with diabetes mellitus and among tuberculosis patients overall. Panel A. Prevalence among individuals with diabetes mellitus. Panel B. Prevalence among tuberculosis patients overall. CI – confidence interval, DM – diabetes mellitus, ES – effect size, PDR-TB – polydrug-resistant tuberculosis, TB – tuberculosis.

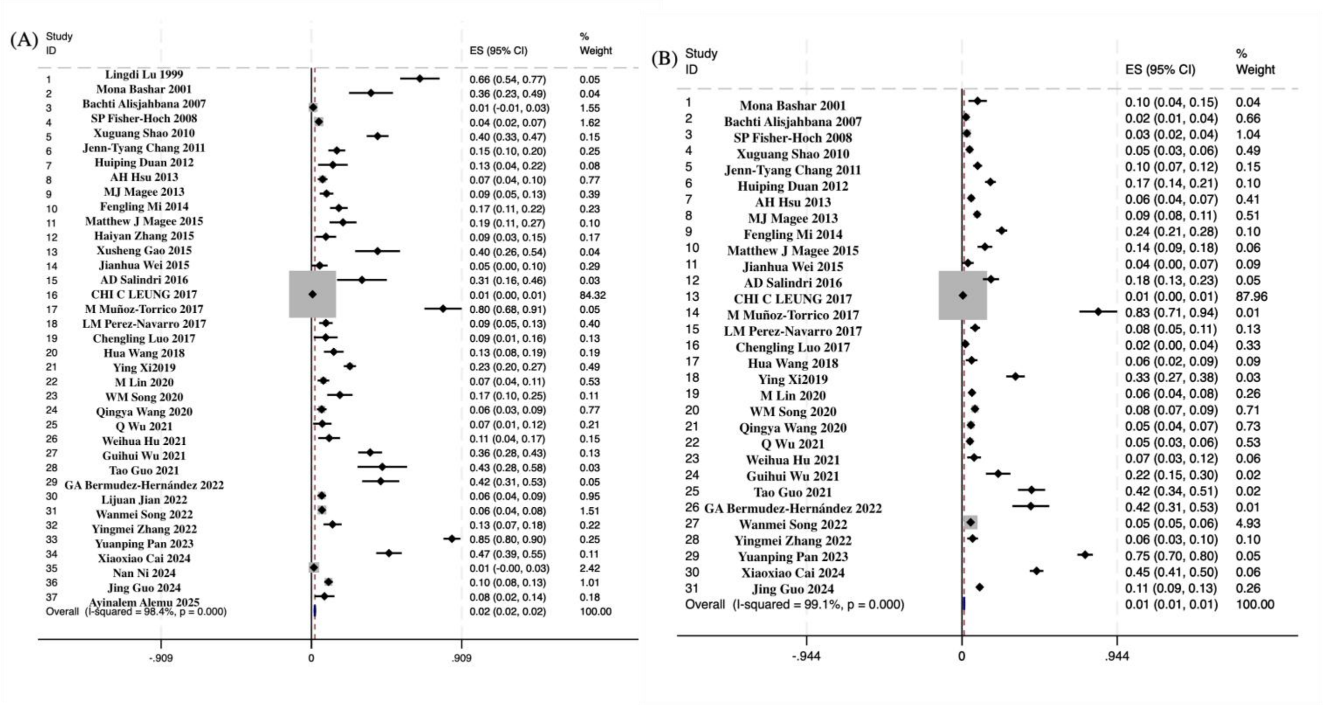

Figure S5. Prevalence of multidrug-resistant tuberculosis among patients with diabetes mellitus and among tuberculosis patients overall. Panel A. Prevalence among individuals with diabetes mellitus. Panel B. Prevalence among tuberculosis patients overall. CI – confidence interval, DM – diabetes mellitus, ES – effect size, MDR-TB – multidrug-resistant tuberculosis, TB – tuberculosis.

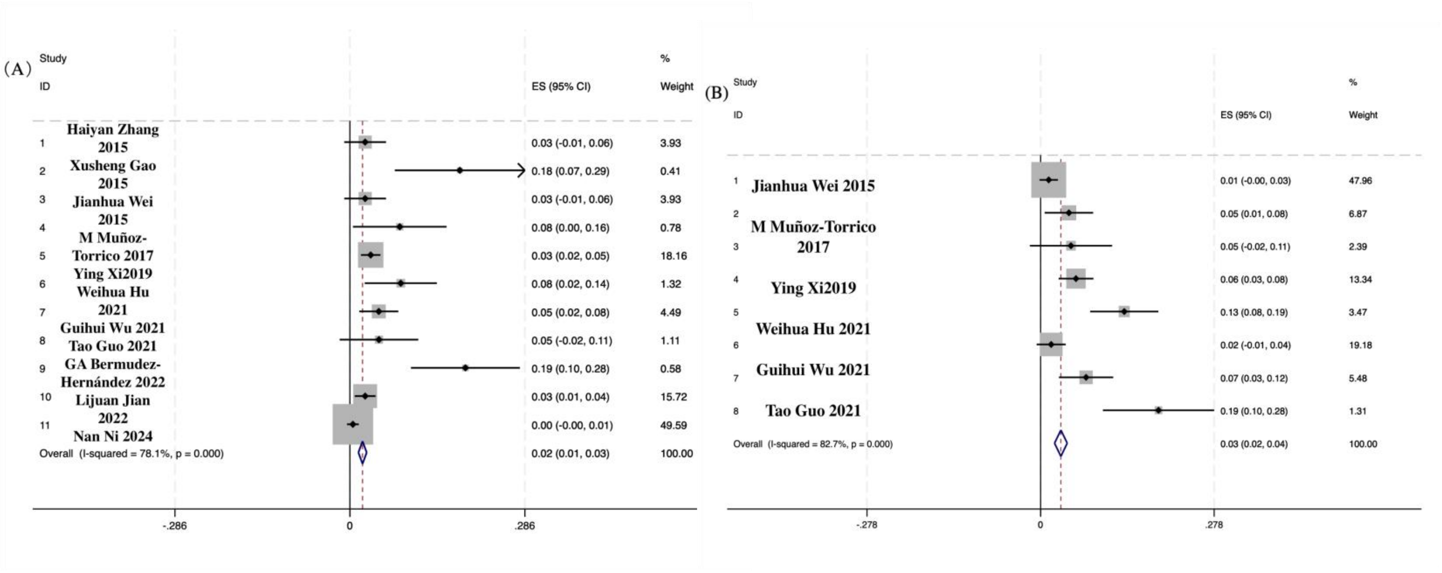

Figure S6. Prevalence of extensively drug-resistant tuberculosis among patients with diabetes mellitus and among tuberculosis patients overall. Panel A. Prevalence among individuals with diabetes mellitus. Panel B. Prevalence among tuberculosis patients overall. CI – - confidence interval, DM – diabetes mellitus, ES – effect size, TB – tuberculosis, XDR-TB – extensively drug-resistant tuberculosis.

Figure S7.Forest plot of the association between glycaemic control and any drug-resistant tuberculosis.

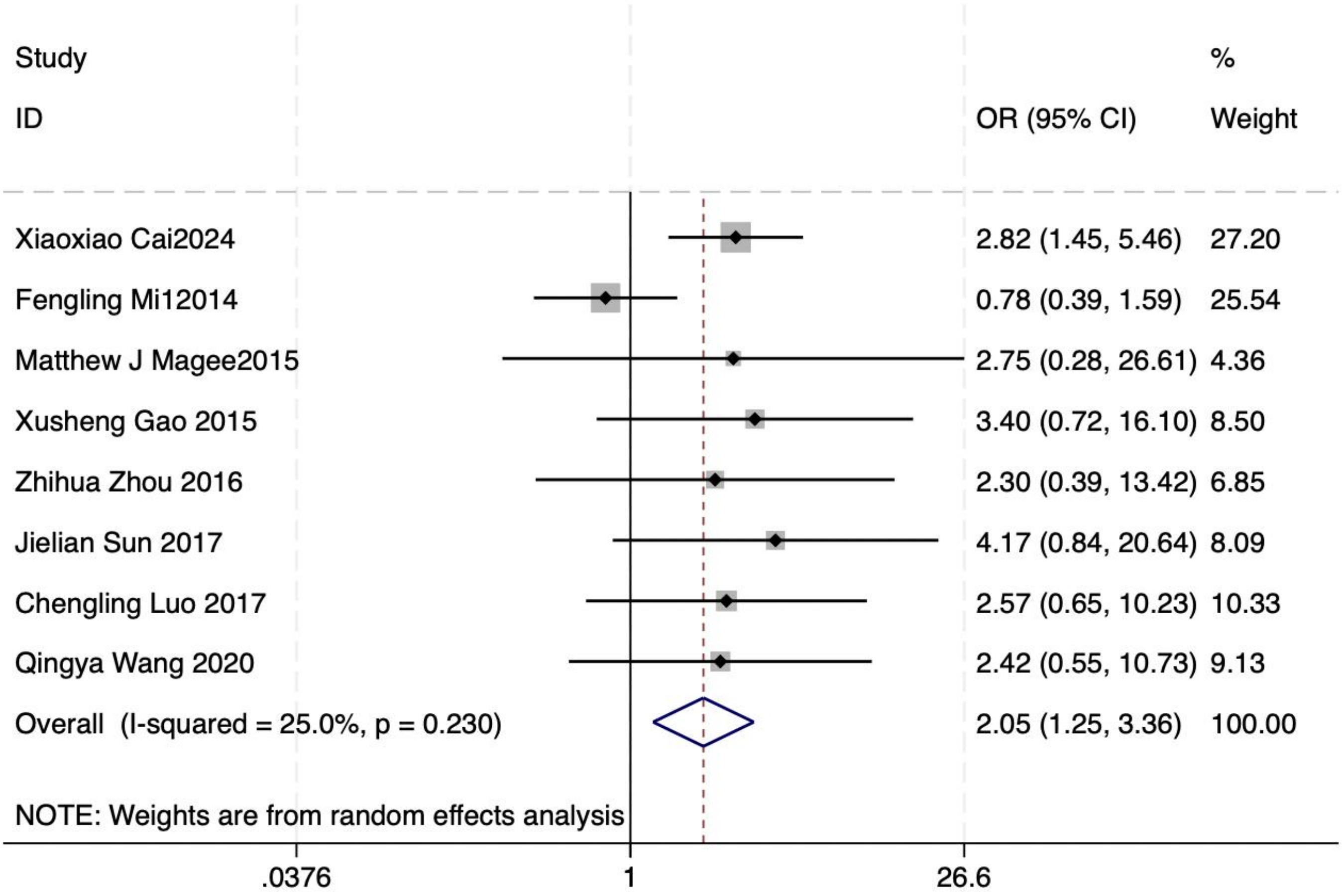

Figure S7.Forest plot of the association between glycaemic control and any drug-resistant tuberculosis.

The forest plot shows study-specific and pooled odds ratios for any drug-resistant tuberculosis comparing patients with poorer glycaemic control to those with better glycaemic control. Poorer glycaemic control was defined as glycated haemoglobin (HbA1c) ⩾ 7.0%, and better glycaemic control was defined as HbA1c < 7.0%. Squares represent study-specific effect estimates, with square size proportional to study weight; horizontal lines indicate 95% confidence intervals; and the diamond represents the pooled random-effects estimate. CI – confidence interval, DR-TB – drug-resistant tuberculosis, HbA1c – glycated haemoglobin, OR – odds ratio.
